# Supplementary material for: Urinary Sodium and Potassium Levels and Blood Pressure in Population with High Sodium Intake
Source: Nutrients. 2020 Nov 10;12(11):3442. doi: 10.3390/nu12113442 (PMC7697285; doi:10.3390/nu12113442)
Supplement: Supplementary file 1 [file nutrients-12-03442-s001.pdf]

Table S1. Baseline characteristics according to the quintiles of sodium-to-creatinine ratio, potassium-to-creatinine ratio or sodium-to potassium ratio in men and women[1]

	Quintile 1	Quintile 2	Quintile 3	Quintile 4	Quintile 5
**Sodium-to-creatinine ratio**					
**Men (n)**	206	207	207	207	207
Sodium-to-creatinine ratio (range)	1.60-9.11	9.12-12.30	12.31-16.05	16.06-20.11	20.13-56.07
Age (years)	36.89 (11.14)	42.25 (13.14)	42.08 (12.18)	45.03 (12.34)	47.49 (14.20)
Sodium (mmol/L)	101.68 (42.58)	123.63 (46.08)	141.03 (48.96)	149.68 (52.61)	157.21 (57.64)
Potassium (mmol/L)	52.70 (28.34)	43.72 (22.06)	42.01 (21.34)	39.32 (19.77)	33.82 (19.39)
BMI (kg/m^2)	24.53 (2.97)	24.37 (2.93)	24.18 (2.84)	24.44 (3.36)	24.50 (3.18)
Smoking status[2]					
Never	61 (29.61)	63 (30.43)	55 (26.57)	38 (18.36)	45 (21.74)
Past	41 (19.90)	61 (29.47)	52 (25.12)	59 (28.50)	74 (35.75)
Current	94 (45.63)	75 (36.23)	89 (43.00)	99 (47.83)	80 (38.65)
Alcohol consumption[2]					
Never	24 (11.65)	30 (14.49)	24 (11.59)	21 (10.14)	32 (15.46)
Past	11 (5.34)	20 (9.66)	15 (7.25)	12 (5.80)	13 (6.28)
Current	171 (83.01)	157 (75.85)	168 (81.16)	174 (84.06)	162 (78.26)
Education level[2]					
Less than high school graduate	20 (9.71)	31 (14.98)	26 (12.56)	39 (18.84)	51 (24.64)
High school graduate	56 (27.18)	66 (31.88)	74 (35.75)	65 (31.40)	80 (38.65)
College or above	129 (62.62)	109 (52.66)	107 (51.69)	103 (49.76)	74 (35.75)
Marital status[2]					
Never married	78 (37.86)	53 (25.60)	54 (26.09)	39 (18.84)	38 (18.36)
Married	125 (60.68)	150 (72.46)	144 (69.57)	164 (79.23)	157 (75.85)
Divorced or widowed or separated	2 (0.97)	3 (1.45)	8 (3.86)	4 (1.93)	11 (5.31)

	Quintile 1	Quintile 2	Quintile 3	Quintile 4	Quintile 5
**Women (n)**	324	323	324	324	324
Sodium-to-creatinine ratio (range)	1.96-11.80	11.82-15.78	15.78-20.34	20.4-27.00	27.05-102.48
Age (years)	38.27 (9.99)	38.80 (10.45)	41.57 (11.94)	43.94 (11.89)	46.98 (13.19)
Sodium (mmol/ L)	100.08 (44.87)	115.33 (49.60)	122.23 (49.42)	128.97 (50.35)	143.33 (53.98)
Potassium (mmol/L)	54.32 (28.45)	42.93 (21.13)	38.66 (19.56)	38.56 (19.68)	34.28 (17.39)
BMI (kg/m^2)	22.64 (3.39)	22.4 (2.99)	22.77 (3.11)	22.95 (3.10)	23.45 (3.13)
Smoking status[2]					
Never	294 (90.74)	291 (90.09)	290 (89.51)	299 (92.28)	283 (87.35)
Past	10 (3.09)	14 (4.33)	14 (4.32)	9 (2.78)	12 (3.70)
Current	18 (5.56)	17 (5.26)	20 (6.17)	15 (4.63)	27 (8.33)
Alcohol consumption[2]					
Never	94 (29.01)	98 (30.34)	123 (37.96)	115 (35.49)	127 (39.20)
Past	35 (10.80)	43 (13.31)	24 (7.41)	39 (12.04)	21 (6.48)
Current	194 (59.88)	182 (56.35)	177 (54.63)	170 (52.47)	176 (54.32)
Education level[2]					
Less than high school graduate	39 (12.04)	52 (16.10)	68 (20.99)	88 (27.16)	114 (35.19)
High school graduate	110 (33.95)	120 (37.15)	104 (32.10)	112 (34.57)	114 (35.19)
College or above	174 (53.70)	149 (46.13)	151 (46.60)	124 (38.27)	95 (29.32)
Marital status[2]					
Never married	79 (24.38)	76 (23.53)	66 (20.37)	34 (10.49)	36 (11.11)
Married	229 (70.68)	222 (68.73)	230 (70.99)	260 (80.25)	237 (73.15)
Divorced or widowed or separated	16 (4.94)	24 (7.43)	28 (8.64)	30 (9.26)	48 (14.81)
Menopause status[2]					
Post menopause	52 (16.05)	56 (17.34)	89 (27.47)	111 (34.26)	138 (42.59)
Pre-menopause	266 (82.10)	264 (81.73)	231 (71.30)	205 (63.27)	174 (53.70)

	Quintile 1	Quintile 2	Quintile 3	Quintile 4	Quintile 5
**Potassium-to-creatinine ratio**					
**Men (n)**	206	207	208	203	210
Potassium-to-creatinine ratio (range)	1.20-2.85	2.85-3.65	3.66-4.44	4.45-5.62	5.66-17.08
Age (years)	35.88 (10.79)	40.50 (12.03)	43.80 (12.48)	45.47 (12.75)	48.06 (13.88)
Sodium (mmol/L)	131.84 (51.39)	131.97 (53.91)	135.87 (55.75)	130.65 (52.55)	142.85 (53.81)
Potassium (mmol/L)	31.23 (15.93)	37.43 (17.89)	40.39 (19.29)	46.28 (25.47)	56.04 (27.37)
BMI (kg/m^2)	24.6 (3.20)	24.42 (2.87)	24.67 (2.93)	24.33 (2.98)	24.00 (3.26)
Smoking status[2]					
Never	58 (28.16)	45 (21.74)	61 (29.33)	42 (20.69)	56 (26.67)
Past	42 (20.39)	55 (26.57)	53 (25.48)	76 (37.44)	61 (29.05)
Current	103 (50.00)	100 (48.31)	82 (39.42)	73 (35.96)	79 (37.62)
Alcohol consumption[2]					
Never	18 (8.74)	22 (10.63)	28 (13.46)	29 (14.29)	34 (16.19)
Past	6 (2.91)	14 (6.76)	19 (9.13)	17 (8.37)	15 (7.14)
Current	182 (88.35)	171 (82.61)	161 (77.40)	157 (77.34)	161 (76.67)
Education level[2]					
Less than high school graduate	22 (10.68)	27 (13.04)	35 (16.83)	39 (19.21)	44 (20.95)
High school graduate	68 (33.01)	76 (36.71)	60 (28.85)	63 (31.03)	74 (35.24)
College or above	115 (55.83)	104 (50.24)	113 (54.33)	101 (49.75)	89 (42.38)
Marital status[2]					
Never married	82 (39.81)	52 (25.12)	51 (24.52)	37 (18.23)	40 (19.05)
Married	116 (56.31)	149 (71.98)	150 (72.12)	162 (79.80)	163 (77.62)
Divorced or widowed or separated	6 (2.91)	6 (2.90)	7 (3.37)	3 (1.48)	6 (2.86)
**Women (n)**	323	324	324	325	323
Potassium-to creatinine ratio (range)	1.44-4.06	4.06-5.16	5.16-6.35	6.35-8.12	8.12-30.17
Age (years)	36.83 (10.44)	39.09 (10.40)	41.70 (11.40)	44.26 (11.72)	47.69 (12.75)

	Quintile 1	Quintile 2	Quintile 3	Quintile 4	Quintile 5
Sodium (mmol/ L)	120.49 (51.05)	122.01 (52.32)	119.19 (48.77)	121.22 (51.15)	127.05 (54.98)
Potassium (mmol/L)	32.78 (16.66)	38.57 (19.48)	40.79 (22.27)	43.56 (21.25)	53.05 (27.15)
BMI (kg/m^2)	22.58 (3.02)	22.85 (3.36)	22.56 (3.10)	23.05 (3.15)	23.16 (3.12)
Smoking status[2]					
Never	283 (87.62)	287 (88.58)	292 (90.12)	307 (94.46)	288 (89.16)
Past	12 (3.72)	16 (4.94)	14 (4.32)	6 (1.85)	11 (3.41)
Current	26 (8.05)	21 (6.48)	16 (4.94)	12 (3.69)	22 (6.81)
Alcohol consumption[2]					
Never	87 (26.93)	96 (29.63)	108 (33.33)	133 (40.92)	133 (41.18)
Past	34 (10.53)	38 (11.73)	37 (11.42)	24 (7.38)	29 (8.98)
Current	202 (62.54)	190 (58.64)	179 (55.25)	168 (51.69)	160 (49.54)
Education level[2]					
Less than high school graduate	40 (12.38)	46 (14.20)	75 (23.15)	85 (26.15)	115 (35.60)
High school graduate	107 (33.13)	118 (36.42)	111 (34.26)	111 (34.15)	113 (34.98)
College or above	176 (54.49)	159 (49.07)	135 (41.67)	128 (39.38)	95 (29.41)
Marital status[2]					
Never married	92 (28.48)	78 (24.07)	52 (16.05)	35 (10.77)	34 (10.53)
Married	211 (65.33)	223 (68.83)	242 (74.69)	254 (78.15)	248 (76.78)
Divorced or widowed or separated	19 (5.88)	23 (7.10)	30 (9.26)	35 (10.77)	39 (12.07)
Menopause status[2]					
Post menopause	43 (13.31)	51 (15.74)	87 (26.85)	113 (34.77)	152 (47.06)
Pre-menopause	278 (86.07)	262 (80.86)	231 (71.30)	207 (63.69)	162 (50.15)
**Sodium-to-potassium ratio**					
**Men (n)**	206	207	207	204	210
Sodium-to-potassium ratio (range)	0.32-2.29	2.30-3.02	3.02-3.89	3.89-4.97	5.00-13.42
Age (years)	40.69 (12.50)	43.30 (13.38)	45.44 (13.18)	42.26 (13.20)	42.06 (12.90)

	Quintile 1	Quintile 2	Quintile 3	Quintile 4	Quintile 5
Sodium (mmol/L)	107.33 (48.79)	126.58 (47.85)	141.75 (50.22)	139.61 (49.74)	157.72 (57.51)
Potassium (mmol/L)	65.63 (30.56)	47.89 (18.31)	41.65 (14.63)	31.59 (11.35)	24.98 (9.89)
BMI (kg/m^2)	24.57 (3.18)	24.42 (2.87)	24.04 (2.68)	24.23 (3.26)	24.74 (3.24)
Smoking status[2]					
Never	59 (28.64)	64 (30.92)	48 (23.19)	52 (25.49)	39 (18.57)
Past	54 (26.21)	49 (23.67)	64 (30.92)	63 (30.88)	57 (27.14)
Current	80 (38.83)	83 (40.10)	80 (38.65)	85 (41.67)	109 (51.90)
Alcohol consumption[2]					
Never	29 (14.08)	23 (11.11)	27 (13.04)	29 (14.22)	23 (10.95)
Past	15 (7.28)	15 (7.25)	20 (9.66)	13 (6.37)	8 (3.81)
Current	162 (78.64)	169 (81.64)	160 (77.29)	162 (79.41)	179 (85.24)
Education level[2]					
Less than high school graduate	24 (11.65)	34 (16.43)	33 (15.94)	38 (18.63)	38 (18.10)
High school graduate	54 (26.21)	67 (32.37)	74 (35.75)	72 (35.29)	74 (35.24)
College or above	128 (62.14)	106 (51.21)	100 (48.31)	94 (46.08)	98 (46.67)
Marital status[2]					
Never married	65 (31.55)	53 (25.60)	38 (18.36)	50 (24.51)	56 (26.67)
Married	138 (66.99)	152 (73.43)	166 (80.19)	143 (70.10)	145 (69.05)
Divorced or widowed or separated	3 (1.46)	2 (0.97)	3 (1.45)	11 (5.39)	9 (4.29)
**Women (n)**	323	324	324	324	324
Sodium-to- potassium ratio (range)	0.43-2.13	2.14-2.76	2.77-3.45	3.45-4.44	4.44-11.60
Age (years)	41.58 (11.34)	41.48 (11.26)	42.48 (12.77)	42.98 (12.54)	41.06 (11.91)
Sodium (mmol/L)	96.53 (45.19)	114.29 (46.53)	123.89 (49.20)	134.78 (51.23)	140.39 (54.11)
Potassium (mmol/L)	62.40 (29.65)	46.69 (18.94)	39.77 (15.91)	34.71 (13.30)	25.24 (10.92)
BMI (kg/m^2)	22.70 (3.28)	22.61 (3.15)	22.65 (2.97)	23.20 (3.27)	23.04 (3.10)
Smoking status[2]					

	Quintile 1	Quintile 2	Quintile 3	Quintile 4	Quintile 5
Never	296 (91.64)	296 (91.36)	290 (89.51)	294 (90.74)	281 (86.73)
Past	12 (3.72)	9 (2.78)	13 (4.01)	13 (4.01)	12 (3.70)
Current	14 (4.33)	17 (5.25)	21 (6.48)	15 (4.63)	30 (9.26)
Alcohol consumption[2]					
Never	116 (35.91)	101 (31.17)	118 (36.42)	120 (37.04)	102 (31.48)
Past	32 (9.91)	36 (11.11)	35 (10.80)	29 (8.95)	30 (9.26)
Current	174 (53.87)	187 (57.72)	171 (52.78)	175 (54.01)	192 (59.26)
Education level[2]					
Less than high school graduate	63 (19.50)	67 (20.68)	76 (23.46)	85 (26.23)	70 (21.60)
High school graduate	109 (33.75)	116 (35.80)	113 (34.88)	98 (30.25)	124 (38.27)
College or above	150 (46.44)	138 (42.59)	135 (41.67)	141 (43.52)	129 (39.81)
Marital status[2]					
Never married	57 (17.65)	61 (18.83)	55 (16.98)	59 (18.21)	59 (18.21)
Married	240 (74.30)	243 (75.00)	232 (71.60)	230 (70.99)	233 (71.91)
Divorced or widowed or separated	26 (8.05)	20 (6.17)	37 (11.42)	33 (10.19)	30 (9.26)
Menopause status[2]					
Post menopause	88 (27.24)	77 (23.77)	103 (31.79)	104 (32.10)	74 (22.84)
Pre-menopause	229 (70.90)	239 (73.77)	215 (66.36)	216 (66.67)	241 (74.38)

[1]Values are means (SD) or number (percentage).
[2]A few participants had missing values.

Table S2. Multivariate-adjusted geometric means (95% CIs)[1] of systolic and diastolic blood pressures (mmHg) according to urinary sodium-to-creatinine, potassium-to-creatinine, or sodium-to-potassium ratios in men and women

	Quintile 1	Quintile 2	Quintile 3	Quintile 4	Quintile 5	*P trend*
**Sodium-to-creatinine ratio**[2]						
**Men**						
Range	1.6-9.1	9.1-12.3	12.3-16.1	16.1-20.1	20.1-56.1	
SBP	118.7 (115.8-121.6)	118.1 (115.2-121.1)	121.1 (118.4-123.9)	119.1 (116.0-122.2)	121.0 (118.4-123.7)	0.09
DBP	73.9 (72.3-75.6)	74.5 (72.6-76.5)	75.7 (73.8-77.6)	75.0 (73.1-77.0)	75.6 (73.8-77.3)	0.13
**Women**						
Range	2.0-11.8	11.8-15.8	15.8-20.3	20.4-27.0	27.1-102.5	
SBP	109.2 (106.8-111.7)	109.4 (107.1-111.9)	109.4 (107.2-111.6)	109.9 (107.5-112.3)	111.8 (109.5-114.1)	0.01
DBP	70.2 (68.7-71.8)	69.5 (68.0-71.1)	69.8 (68.3-71.4)	69.4 (67.8-71.0)	71.3 (69.8-72.9)	0.05
**Potassium-to-creatinine ratio**[3]						
**Men**						
Range	1.2-2.9	2.9-3.7	3.7-4.4	4.5-5.6	5.7-17.1	
SBP	121.0 (118.0-124.1)	119.3 (116.5-122.1)	120.1 (117.3-122.9)	119.3 (116.8-121.9)	118.8 (115.9-121.6)	0.21
DBP	75.1 (73.2-77.1)	75.2 (73.4-77.1)	75.3 (73.6-77.0)	74.3 (72.7-76.0)	74.6 (72.7-76.6)	0.46
**Women**						
Range	1.4-4.1	4.1-5.2	5.2-6.4	6.4-8.1	8.1-30.2	
SBP	110.7 (108.1-113.3)	110.8 (108.5-113.0)	109.7 (107.4-112.0)	111.2 (108.8-113.7)	108.8 (106.6-111.2)	0.13
DBP	70.7 (69.0-72.4)	70.8 (69.2-72.4)	69.8 (68.4-71.4)	70.7 (69.1-72.4)	69.2 (67.6-70.8)	0.04
**Sodium-to-potassium ratio**						
**Men**						
Range	0.3-2.3	2.3-3.0	3.0-3.9	3.9-5.0	5.0-13.4	
SBP	116.8 (114.1-119.5)	119.1 (116.3-122.0)	120.0 (117.2-122.9)	121.3 (118.7-124.0)	120.8 (118.0-123.7)	0.002
DBP	72.9 (71.2-74.6)	76.2 (74.4-78.0)	76.0 (74.0-77.9)	75.9 (74.2-77.6)	74.5 (72.6-76.4)	0.39
**Women**						
Range	0.4-2.1	2.1-2.8	2.8-3.5	3.5-4.4	4.4-11.6	
SBP	109.0 (106.8-111.2)	109.3 (107.0-111.7)	110.2 (107.9-112.5)	110.7 (108.2-113.2)	110.9 (108.5-113.3)	0.02
DBP	69.4 (67.9-70.9)	70.4 (68.9-71.9)	70.3 (68.8-71.8)	70.1 (68.4-71.8)	70.5 (68.9-72.1)	0.19

Abbreviation: CI, confidence interval; SBP, systolic blood pressure; DBP, diastolic blood pressure; BMI, body mass index.

[1]Adjusted for age, age^2, BMI (kg/m^2; continuous), pack years of smoking (never, <10, 10 to <20, ≥20 for men and never, <3, 3 to <6, ≥6 for women), alcohol consumption(none, <15, 15 to <30, ≥30 g/day for men and none, <3.75, 3.75 to <7.5, ≥7.5 g/day for women), marriage status (never, married, divorced or widowed or separated), education level (less than high school graduate, high school graduate, college or above), and menopause status (pre, post) for women.

[2]We further adjusted for urinary potassium (mmol/L; continuous) in the model.

[3]We further adjusted for urinary sodium (mmol/L; continuous) in the model.

Table S3. Multivariate-adjusted geometric means (95% CIs)[1] of systolic and diastolic blood pressures (mmHg) according to urinary sodium-to-creatinine and potassium-to-creatinine ratios by eGFR, urinary potassium level, education level, and married status

	n	Quintile1	Quintile2	Quintile3	Quintile4	Quintile5	P_{trend}	$P_{interaction}$
**Sodium-to-creatinine[2]**								
**SBP**								
eGFR (mL/min/1.73 m²)								
<90	1933	113.3 (111.1-115.5)	113.0 (111.2-114.9)	112.8 (111.1-114.6)	113.3 (111.4-115.1)	114.8 (113.0-116.6)	0.07	0.62
≥90	720	114.5 (110.5-118.6)	116.0 (111.8-120.3)	117.1 (113.3-121.1)	115.6 (111.6-119.8)	118.5 (113.9-123.3)	0.05	
Potassium (mmol/L)								
<37	1281	113.8 (113.4-117.3)	113.1 (110.9-115.4)	114.0 (111.8-116.2)	113.8 (111.7-116.0)	115.4 (113.4-117.3)	0.13	0.40
≥37	1372	113.3 (113.3-118.2)	114.1 (112.2-116.1)	114.0 (112.0-116.1)	113.6 (111.4-115.8)	115.7 (113.3-118.2)	0.12	
Education level								
Less than high school graduate	1429	116.6 (116.8-121.0)	117.1 (114.9-119.3)	118.0 (115.8-120.3)	116.9 (114.9-119.0)	118.9 (116.8-121.0)	0.11	0.86
High school graduate or college above	1215	108.3 (108.2-113.9)	109.4 (107.0-111.9)	108.4 (105.8-111.1)	109.8 (107.2-112.3)	111.0 (108.2-113.9)	0.03	
Married status								
Married	2092	114.7 (114.5-117.7)	115.0 (113.3-116.7)	115.5 (113.9-117.1)	115.0 (113.5-116.6)	116.1 (114.5-117.7)	0.20	0.49
Non-married	553	104.1 (103.7-113.3)	105.5 (101.6-109.5)	104.1 (100.1-108.3)	105.2 (101.3-109.1)	108.4 (103.7-113.3)	0.05	
**DBP**								
eGFR (mL/min/1.73 m²)								
<90	1933	72.2 (70.8-73.5)	71.6 (70.3-72.9)	71.5 (70.3-72.8)	71.7 (70.4-73.0)	72.5 (71.2-73.8)	0.34	0.58
≥90	720	72.4 (69.7-75.2)	73.8 (71.0-76.7)	73.9 (71.3-76.7)	73.9 (71.0-76.9)	73.4 (70.0-76.9)	0.32	
Potassium (mmol/L)								
<37	1281	71.9 (71.3-74.2)	71.7 (70.2-73.4)	72.5 (71.0-74.0)	72.5 (71.0-74.0)	72.8 (71.3-74.2)	0.25	0.70
≥37	1372	72.6 (71.7-75.1)	72.9 (71.5-74.2)	72.6 (71.1-74.0)	72.2 (70.7-73.8)	73.4 (71.7-75.1)	0.57	
Education level								
Less than high school graduate	1429	74.5 (73.8-76.6)	74.6 (73.1-76.1)	75.2 (73.7-76.6)	74.1 (72.7-75.5)	75.2 (73.8-76.6)	0.55	0.82

	n	Quintile1	Quintile2	Quintile3	Quintile4	Quintile5	P_{trend}	$P_{interaction}$
High school graduate or college above	1215	69.4 (68.4-72.3)	69.9 (68.2-71.6)	69.2 (67.4-70.9)	70.6 (68.8-72.5)	70.4 (68.4-72.3)	0.22	
Married status								
Married	2092	72.9 (72.2-74.4)	72.9 (71.8-74.1)	73.2 (72.0-74.3)	73.0 (71.9-74.1)	73.3 (72.2-74.4)	0.55	0.92
Non-married	553	69.0 (66.6-74.3)	69.4 (66.3-72.7)	68.5 (65.2-71.9)	69.8 (66.6-73.1)	70.3 (66.6-74.3)	0.37	
**Potassium-to-creatinine**[3]								
**SBP**								
eGFR (mL/min/1.73 m^2)								
<90	1933	114.7 (112.3-117.2)	114.0 (112.1-115.9)	113.7 (112.0-115.5)	113.4 (111.6-115.2)	112.3 (110.5-114.1)	0.02	0.74
≥90	720	116.5 (112.2-121.0)	116.2 (112.1-120.4)	115.9 (111.6-120.2)	118.0 (114.0-122.2)	115.5 (110.6-120.7)	0.94	
Education level								
Less than high school graduate	1429	118.6 (114.5-118.8)	118.2 (115.8-120.5)	117.9 (115.8-120.1)	117.6 (115.4-119.8)	116.6 (114.5-118.8)	0.13	0.44
High school graduate or college above	1215	110.2 (105.3-110.5)	109.3 (106.8-112.0)	109.5 (107.1-111.9)	109.8 (107.4-112.4)	107.8 (105.3-110.5)	0.11	
Married status								
Married	2092	116.1 (112.2-115.4)	116.1 (114.3-117.8)	115.4 (113.9-116.9)	116.1 (114.4-117.7)	113.8 (112.2-115.4)	0.03	0.91
Non-married	553	106.0 (101.6-110.1)	104.3 (100.4-108.5)	106.4 (102.1-110.9)	102.7 (98.6-106.9)	105.8 (101.6-110.1)	0.62	
**DBP**								
eGFR (mL/min/1.73 m^2)								
<90	1933	72.4 (70.8-74.0)	72.2 (71.0-73.5)	72.0 (70.7-73.3)	71.9 (70.7-73.2)	71.1 (69.8-72.3)	0.04	0.62
≥90	720	73.9 (70.8-77.2)	73.7 (70.7-76.9)	72.7 (69.9-75.7)	74.5 (71.9-77.2)	72.9 (69.2-76.8)	0.62	
Education level								
Less than high school graduate	1429	75.5 (72.5-75.3)	75.1 (73.6-76.7)	74.7 (73.2-76.1)	75.0 (73.5-76.4)	73.9 (72.5-75.3)	0.06	0.82
High school graduate or college above	1215	70.2 (67.1-70.8)	70.0 (68.2-71.9)	69.7 (68.1-71.4)	70.2 (68.5-71.8)	68.9 (67.1-70.8)	0.21	
Married status								
Married	2092	116.1 (112.2-115.4)	116.1 (114.3-117.8)	115.4 (113.9-116.9)	116.1 (114.4-117.7)	113.8 (112.2-115.4)	0.03	0.87
Non-married	553	106.0 (101.6-110.1)	104.3 (100.4-108.5)	106.4 (102.1-110.9)	102.7 (98.6-106.9)	105.8 (101.6-110.1)	0.62	

Abbreviation: CI, confidence interval; SBP, systolic blood pressure; DBP, diastolic blood pressure; BMI, body mass index.

[1]Adjusted for age, age^2, BMI (kg/m^2; continuous), pack years of smoking (never, <10, 10 to <20, ≥20 for men and never, <3, 3 to <6, ≥6 for women), alcohol consumption(none, <15, 15 to <30, ≥30 g/day for men and none, <3.75, 3.75 to <7.5, ≥7.5 g/day for women), marriage status (never, married, divorced or widowed or separated), education level (less than high school graduate, high school graduate, college or above), and menopause status (pre, post) for women.
[2]We further adjusted for urinary potassium (mmol/L; continuous) in the model.
[3]We further adjusted for urinary sodium (mmol/L; continuous) in the model.

Table S4. Multivariate-adjusted geometric means (95% CIs)[1] of systolic and diastolic blood pressures (mmHg) according to sodium contributing food score by BMI category

	Quintile 1	Quintile 2	Quintile 3	Quintile 4	Quintile 5	P trend
All						
BMI<25 kg/m^2						
SBP	111.9 (110.0-114.3)	111.2 (109.3-113.1)	112.5 (110.7-114.3)	111 .0 (109.2-112.8)	112.1 (110.0-114.3)	0.94
DBP	71.1 (69.7-72.5)	70.5 (69.2-71.8)	71.0 (69.7-72.3)	70.6 (69.4-71.9)	71.1 (69.7-72.5)	0.90
BMI≥25 kg/m^2						
SBP	117.8 (117.4-123.8)	116.7 (114.0-119.5)	119.6 (116.5-122.8)	120.4 (117.1-123.8)	120.6 (117.4-123.8)	0.10
DBP	74.8 (74.2-78.8)	74.5 (72.5-76.6)	77.1 (74.9-79.3)	76.2 (73.7-78.7)	76.5 (74.2-78.8)	0.17
Men						
BMI<25 kg/m^2						
SBP	114.7 (113.2-121.4)	114.7 (111.4-118.1)	116.8 (113.5-120.3)	117.0 (114.0-120.1)	117.2 (113.2-121.4)	0.15
DBP	71.4 (70.9-76.4)	71.5 (69.5-73.6)	73.2 (70.9-75.5)	72.1 (70.0-74.3)	73.6 (70.9-76.4)	0.20
BMI≥25 kg/m^2						
SBP	108.0 (105.0-110.9)	107.2 (104.7-109.8)	106.6 (104.1-109.1)	107.1 (104.5-109.8)	107.9 (105.0-110.9)	0.78
DBP	68.6 (66.9-70.8)	68.8 (66.9-70.7)	68.2 (66.4-70.0)	68.2 (66.4-70.1)	68.8 (66.9-70.8)	0.99
Women						
BMI<25 kg/m^2						
SBP	123.6 (121.7-132.6)	124.6 (119.4-130.0)	122.9 (117.7-128.4)	125.1 (119.7-130.8)	127.1 (121.7-132.6)	0.29
DBP	77.9 (76.6-83.6)	78.2 (74.9-81.5)	77.9 (74.6-81.4)	79.3 (75.8-83.0)	80.0 (76.6-83.6)	0.35
BMI≥25 kg/m^2						
SBP	115.0 (113.8-125.2)	118.1 (112.2-124.3)	121.8 (114.6-129.5)	118.4 (112.5-124.6)	119.4 (113.8-125.2)	0.11
DBP	72.3 (71.4-79.3)	74.5 (71.0-78.2)	77.0 (72.6-81.6)	74.9 (71.5-78.4)	75.2 (71.4-79.3)	0.15

Abbreviation: CI, confidence interval; BMI, body mass index; SBP, systolic blood pressure; DBP, diastolic blood pressure.

[1]Adjusted for age, age^2, BMI (kg/m^2; continuous), pack years of smoking (never, <10, 10 to <20, ≥20 for men and never, <3, 3 to <6, ≥6 for women), alcohol consumption(none, <15, 15 to <30, ≥30 g/day for men and none,<3.75, 3.75 to <7.5, ≥7.5 g/day for women), marriage status (never, married, divorced or widowed or separated), education level (less than high school graduate, high school graduate, college or above), menopause status (pre, post) for women, and urinary potassium (mmol/L; continuous)

Table S5. Factor loading values of food groups contributing to urinary potassium-to-creatinine ratio through reduced rank regression analysis

Food groups	Factor loadings[1]
Possitive associations	
Vegetables	+0.265
Potato and sweet potato	+0.249
Fruit	+0.233
Inverse associations	
Ramen	-0.421
Soft drink	-0.271
Poultry	-0.271
Red meat and beef soup	-0.249
Processed meat	-0.246
Noodle	-0.230
Pizza	-0.230

[1]The food groups with the absolute value of factor loading greater than 0.20 were selected.

Table S6. Multivariate-adjusted geometric means (95% CIs)[1] of systolic and diastolic blood pressures (mmHg) according to potassium-contributing food score[1]

	Quintile 1	Quintile 2	Quintile 3	Quintile 4	Quintile 5	*P* trend
All						
SBP	113.9 (112.3-115.5)	113.0 (111.4-114.6)	114.5 (113.0-116.0)	113.8 (112.2-115.4)	114.6 (112.8-116.3)	0.47
DBP	72.3 (71.2-73.5)	71.9 (70.8-73.0)	72.7 (71.6-73.8)	72.4 (71.3-73.5)	72.8 (71.6-73.9)	0.50
Men						
SBP	118.8 (116.0-121.6)	118.9 (116.1-121.8)	119.4 (116.6-122.3)	120.3 (117.6-123.1)	121.4 (118.2-124.7)	0.11
DBP	74.3 (72.4-76.2)	74.4 (72.6-76.2)	75.1 (73.3-77.0)	74.9 (73.1-76.7)	76.4 (74.3-78.5)	0.16
Women						
SBP	109.5 (107.1-112.0)	109.4 (107.1-111.7)	109.5 (107.2-111.9)	109.1 (106.7-111.6)	110.1 (107.5-112.7)	0.71
DBP	69.6 (68.0-71.3)	70.0 (68.4-71.7)	70.0 (68.4-71.7)	69.6 (68.0-71.2)	70.2 (68.5-71.9)	0.60

Abbreviation: CI, confidence interval; SBP, systolic blood pressure; DBP, diastolic blood pressure

[1]Adjusted for age, age^2, BMI (kg/m^2; continuous), pack years of smoking (never, <10, 10 to <20, ≥20 for men and never, <3, 3 to <6, ≥6 for women), alcohol consumption(none, <15, 15 to <30, ≥30 g/day for men and none, <3.75, 3.75 to <7.5, ≥7.5 g/day for women), marriage status (never, married, divorced or widowed or separated), education level (less than high school graduate, high school graduate, college or above), menopause status (pre, post) for women, total energy intake (kcal/d; continuous) and urinary sodium (mmol/L; continuous).
